# In the need of a digital cicerone in healthcare? – Guidance for parents

**DOI:** 10.1186/s12884-022-05120-0

**Published:** 2022-11-23

**Authors:** C Bäckström, R Knez, M Fahlgren, M Synnergren, V Larsson

**Affiliations:** 1 School of Health Sciences, University of Skövde, SE-541 28 Skövde, Sweden; 2 Department of Caring Science, University of Borås, SE-501 90 Borås, Sweden; 3 Skaraborg Hospital, Lövängsvägen, SE-541 42 Skövde, Sweden

**Keywords:** Pregnancy, Labor, Birth, Parenthood, Digitalization, Antenatal care, Child healthcare

## Abstract

**Objective:**

To explore parents’ experiences using digital tools in relation to pregnancy, labor and birth, and the child’s first 18 months.

**Background:**

Parents find relevant information using digital healthcare tools, material obtained from professionals, as well as personal opinions and experiences that vary in quality.

**Method:**

Fifteen parents were interviewed and data were analyzed beginning with content analysis and followed by thematic analysis.

**Results:**

The main theme was *insecurity and responsibility for own choices and knowledge.* Parents use digital tools to take responsibility for their insecurity and need for knowledge when entering parenthood.

**Conclusion:**

The parents’ experiences highlighted that (1) insecurity can be both eased and enhanced using digital tools, (2) they took responsibility for feelings of insecurity and the search for knowledge, and (3) they needed knowledge to make the right choices and feel secure that these choices are made in the best interest of their new family.

## Introduction

Over the last decade, and especially since the outbreak of coronavirus disease 2019 (COVID-19), digital tools*—*as an umbrella term for different technological sources, such as applications, websites, podcasts, forums, social media groups, and specialized professional websites*—*have been increasingly used in health care [1], and the field of obstetrics is no exception [2]. For example, digital parental support tools have been proposed as a complement to standard antenatal care [3, 4]. However, the use of these digital tools does not automatically imply that healthcare professionals have answers to some important questions, such as *What kind of support are expecting parents searching for?* and *What needs during pregnancy and early parenting periods do parents really have?* For example, midwives in antenatal care have generally ambivalent views on the use of digital technology. On the one hand, they describe benefits (i.e., modern antenatal care with opportunities for expecting mothers to make informed decisions); on the other, they describe limitations and risks (i.e., conveys unreliable information and has negative impacts on the relationship between midwives and expecting mothers) [5].

While accessing relevant information using digital tools, parents may find, on the one hand, material obtained from professional sources, and, on other, personal opinions and experiences that may vary in quality. Furthermore, digital tools are not just a source of information that should provide support when concerns arise. They might serve higher purposes just fulfilling basic needs for parental support. For example, with a technological design that accounts for and resolves discrepancies in communication between parent and child, and the complex emotions attributable to parents’ daily life schedules, digital tools may support parent–child relations [2]. Therefore, digital tools may provide a virtual space that invites future parents to “weave together an unusually fine fabric that will embrace their children, a matrix or a nest in which the newborn can then land softly, warmly and safely” ( [6], p 7). This metaphor is more than just an illustration of interpersonal space between two adults; it may also serve as a metaphor for their own transition to parental roles. Importantly, this matrix must be built on a solid foundation of not only the perceived quality of a parental couple’s relationship and social support but also professional guidance. In order for digital tools to be able to ensure this higher level of meaning, they must first be capable of comprehending expecting parents’ fundamental needs. To broaden knowledge about such needs, further research is required.

Expecting parents, especially first-time parents, sometimes undergo a delicate and sensitive process of adaptation to changes that accompany parenting. Feelings of insecurity are an understandable phenomenon that also accompanies the life changes related to parental transition [7]. Changes occur at different paces, and everyone has a different capacity to quickly adapt to new situations. Mercer [8] described, in a theoretical model, adaptation to the maternal role as a multi-phased and leveled process that may be hindered by poor health, for example. Pregnancy is a temporally limited period and, considered as the parents’ preparation time, it can sometimes be even shorter than expected (as in cases of premature birth). Just as well, during the COVID-19 pandemic, women have described their pregnancy experiences as “in the shadow of the unknown,” balancing an information echo and social isolation [9], which is alarming as expectant parents need to learn from those in their social network, such as peers and/or other new parents [10]. Therefore, certain external pressures regarding limited (available) time or social contextual circumstances for adaptation are unavoidable.

No matter how well expecting parents prepare themselves, pregnancy, birth, and parenting may easily be perceived as beyond their control. Unsurprisingly, therefore, concerns may be almost constantly present during pregnancy but also during early parenthood. These concerns may represent a whole spectrum of worries—from restlessness, insecurity, and hesitation to fear, anxiety, and panic—that might significantly influence expecting parents’ lives [11].

During pregnancy, expecting parents face many, sometimes small, choices. However, ultimately, each of these small choices may be greatly important to the outcome of the pregnancy and, later, the health/welfare of a child or mother. Accordingly, expecting parents are likely to question themselves. For instance, they might wonder, *“Is how I feel okay, or should I do something about it? Should I act on my feelings and seek care?”* When first-time expecting parents especially cannot relate or compare their pregnancy experiences with familiar situations, they might request information ahead of time concerning possible changes in their relationship and related coping strategies [10]. Parents may feel a heavy burden of responsibility alongside an awareness that some of their choices may be completely wrong; in the worst-case scenario, such wrong decisions could even result in illness or death. Moreover, first-time parents usually lack relevant knowledge as well as experience, so their worries, anxieties, and fears are unsurprising. They must grapple with not only knowledge gaps but also the lack of a frame of reference. In moments of insecurity, unable to rely on their own knowledge, they might feel an urge to seek guidance, and such guidance may be sought via digital tools [4, 12]. Digital tools that allow expecting mothers to ask questions in various parenting forums could facilitate their feelings of calmness [13] and reduce feelings of depression or anxiety [14]. However, such a search may not be positively experienced by all expecting parents. Research has shown, for example, that lesbian, gay, bisexual, transgender, and queer (LGBTQ) persons may find online forums to be oppressive or exclusive when navigating pregnancy-related issues [15].

### Theoretical Approach

#### Transition theory

Adaptation to life changes, and to health transitions, has been theorized within transition theory in health [11]. Transition theory as used by, for example, Meleis et al. [11], explores the meaning of transitory processes for those undergoing them, as follows, “Change may be related to critical or disequilibrating events, to disruptions in relationships and routines, or to ideas, perceptions, and identities” ( [11], p 20). Change in transition theory refer to community and societal conditions, with a focus on the transitioning of persons.

To theoretically approach parents’ experiences of digital tools and search for guidance concerning transitory processes and practices, through variations of insecurity, of the unknown, and of responsibility of social structures known and enacted, a discussion employing the concepts of liminality and communitas will be attempted and compared to parental transition theory.

#### The concepts of liminality and communitas

The concepts of liminality and communitas have been developed within the social science field [16], although the possibility of their transfer to other fields of research has been questioned [17]. However, these concepts have been applied recently within nursing [18], education [19], and psychology [20], and have even been applied to phenomena such as the current COVID-19 pandemic [21, 22] and the intersections of parenthood, gender, and digital, social worlds [23].

Liminality, for example, as discussed from the perspectives of van Gennep and Turner [16], is the middle of a ritual process in which persons are separated from everyday social structures and undergo a phase of change: liminality [16]. The word *anti-structure* has been used to describe the otherness of the phase, but the transferred meaning of anti-structure outside religious practice has been debated [17]. In later research, liminality has been described as “ambiguity and disorientation” ( [21], p 81), uncertainty, status, ambiguity in relation to structure and communitas, obedience [18], disequilibrium [19], or as a transformative space within arts and music [20].

The concept of communitas [16] reads as a group of persons who share a social bond and friendship and have a common experience of liminality, undergone together, or, as phrased by Buechner et al. ( [19], p 87), “a shared feeling” and a “sense of belonging.” The members of the communitas are, after the liminality, reincorporated into the social structure, into norms and social positions, with an acceptance and embodiment of the new role and status bestowed upon them by the passage [16].

Liminality and communitas may serve as concepts of parental passage from being two partners, secure in their identities as individuals, to being parents and sharing a familial responsibility, via the transformation of pregnancy and childbirth, for whom a possible digital communitas could be explored.

To broaden knowledge, further research is needed on parents’ experiences that can in turn be utilized in the future planning and development of digital tools for parental support. By interviewing parents themselves, we can provide professionals with additional knowledge that, in turn, may help them guide expecting and new parents through the exponentially growing field of digital resources. By addressing parents’ real experiences, and problematizing them from a theoretical perspective, healthcare professionals may be better able to comprehend parents’ needs and provide appropriate guidance. Therefore, the aim of this study was to explore parents’ experiences using digital tools in relation to pregnancy, labor and birth, and the child’s first 18 months.

## Methods

This study was part of a larger research project, *“Digital parental support,”* which includes explorative and mixed methods studies and inductive and deductive approaches. Within the larger research project, different interventions, including digital parental support developed as serious games, will be explored [6]. The present study comprised one part of a preliminary study aimed at broadening knowledge about digital parental support. The study employed an inductive qualitative design with semi-structured interviews to record parents’ experiences using digital tools in relation to pregnancy, labor and birth, and the child’s first 18 months.

### Setting and sample

The current study was conducted in two regions in southwestern Sweden. These regions are home to approximately 15,000 residents. Recruitment via convenience sampling was carried out between autumn 2019 and winter 2020. The recruited participants were also involved in another research project, *Reinforced Parenting-Extended Home Visits* [24]. For inclusion in the study, the participants had to live in one of the two regions and had to be either expecting a child or have had a child in the previous 18 months. Potential participants were asked whether they were interested in participating in the study by child healthcare (CHC) nurses. In total, 15 parents, ranging in age from 23 to 57, agreed to participate (11 mothers and 4 fathers). Most of the participants (11) spoke Swedish, while the rest spoke Arabic or English and were from other European or African countries. Among the participants, nine had a high school education. Eleven participants were either expecting a child or had a child younger than 18 months, whereas four participants had another child between one and seven years old. Some of the participants participated individually, whereas others participated as a parental couple.

### Data collection

After written approval by the responsible managers to conduct the study at the CHC centers, the recruitment process was initiated by the CHC nurses. Parents interested in participating contacted the researchers to receive further information about the study and to arrange a time for an interview. The interviews followed a semi-structured interview guide, including open-ended and follow-up questions, and were tested in three pilot interviews to ensure clarity and comprehensibility for the interviewees. The results from these pilot interviews demonstrated that the interview questions were easy to understand and respond to, and therefore no adjustments needed to be made. The pilot interviewees met the inclusion criteria (except that they did not participate in the *Reinforced Parenting-Extended Home Visits* project), and the narratives were well aligned with the aim of the study. Therefore, all three pilot interviews were included in the data analysis in the current study.

At the beginning of each interview, socio-demographic questions were asked, such as *How old are you?* and *How old is your child?* Then, an open-ended question was asked: *What type of digital tools have you been using in regard to pregnancy, labor and birth, and parenthood?* Follow-up questions were also employed, such as *Could you please describe further?* and *How did you experience that?* The interviews were held between November 2019 and January 2020 by three of the authors (CB, MF, MS) at the CHC centers or at the participants’ homes, and lasted between 19 and 61 min. Three of the interviews were held with the parental couple together. Ten interviews were held in the Swedish language with Swedish-speaking participants, one interview was held in English, and the others were held in Arabic with a professional interpreter. All interviews were recorded and transcribed verbatim in Swedish (14 interviews) or English (one interview). The transcripts were outlined on 108 A4 pages with single-line spacing.

### Data analysis

Initially, the material was analyzed through content analysis [25] by two of the authors [MF, MS] in a Master of Science thesis under supervision of the first author [CB]. The initial analysis process resulted in different categories and one preliminary theme, not presented in current study. To deepen the analysis and data comprehension, an iterative process using thematic analysis followed the content analysis. This was carried out by three of the authors [CB, RK, VL], who thematically analyzed and reported patterns (themes) to address the aim of the study. For this, a step-by-step process as described by Braun and Clarke [26] inspired the analysis: *Step 1* - All authors read the transcripts to familiarize themselves with the data; *Step 2 & 3* - Themes of the content analysis were compared and potential themes were further elaborated; *Step 4* - The themes were controlled in relation to the content analysis and overall data; *Step 5* – Themes were defined and named; *Step 6* - Report was written at the same time as themes were controlled with data through a ‘went back and forth’ process. For further details, please see Table 1. Finally, all authors agreed on the themes, and the overall theme, which are presented in the results section of this paper.

**Table 1:** Thematic analysis similar to Braun and Clarke (2008)

Step 1. Familiarizing yourself with the data	All authors read the transcripts to familiarize themselves with the data. Notes were taken for initial ideas about patterns of meaning.
Step 2. Generating initial codes Step 3. Searching for themes	Themes of the content analysis were compared concerning meaning regarding the phenomenon under study. Potential themes were further elaborated to capture a coherent picture of data relevant for each theme.
Step 4. Reviewing themes	The themes were controlled in relation to the content analysis and overall data to generate a thematic map of the analysis.
Step 5. Defining and naming themes	Themes, as well as one overall theme, were defined and named.
Step 6. Producing the report	Report was written at the same time as themes were controlled with data through a ‘went back and forth’ process.

## Results

During the thematic analysis, three subthemes were identified: *insecurity, responsibility*, and *knowledge.* Additionally, one overall theme emerged: *insecurity and responsibility for own choices and knowledge* (Table 2).

**Table 2:** Overview of overall theme and subthemes

Overall theme	Subthemes
Insecurity and responsibility for own choices and knowledge	Insecurity
	Responsibility
	Knowledge

### Insecurity and responsibility for own choices and knowledge

The results revealed various reasons for parents’ use of digital tools, one of which were feelings of insecurity. Another reason was their need for knowledge that would permit them to make their own choices related to both pregnancy, labor, and parenting. A third reason was their wish for emotional support, which could be fulfilled when they had the opportunity to express their emotions to and receive responses from others. They turned to digital tools to search for answers to their questions, and they described that, on the one hand, some questions were answered, whereas, on the other, some questions were not, which may have increased their feelings of insecurity. Their use of digital tools allowed them to take responsibility for both their insecurity and their need for knowledge so that they could make more informed choices in the best interest of themselves, their child, and their family.

Furthermore, the results highlighted individual outcomes from using digital tools. For some parents, their use of digital tools did not always lead to increased knowledge or relief from feelings of insecurity. Indeed, for some, their feelings of insecurity were worsened, especially when the information they obtained was conflicting or perceived as unreliable. The participants’ feelings of insecurity were, most commonly, reduced when they had access to digital tools recommended or controlled by healthcare professionals, such as midwives or CHC nurses. This could be understood as if the participants let the professionals be responsible for the knowledge they gained.

#### Insecurity

The participants mentioned that the digital tools sometimes generated a large amount of unsorted information, which they felt to be overwhelming. They had difficulty determining on which information to focus, which in turn worsened their feelings of insecurity. Just as well, due to their choices, feelings of anxiety arose concerning what types of information they might have missed. The participants described this anxiety as resulting from retaining general information instead of increasing their subjective knowledge. Also, the participants experienced insecurity, frustration, and confusion in attempting to understand which search strategy to use on different digital platforms, which information was relevant to them, and how to understand conflicting information, as one parent described: “*When you search on Google via the web… there can be different answers … 20 answers to one and the same question … very broad … You do lose your concentration on which* [information] *is correct and fits in my case.”*

The participants described concerns about their own or their child’s health, or the well-being of the unborn fetus during pregnancy, as a reason for seeking information via digital tools. The participants felt that their insecurity could worsen while using digital tools, which could result in more intensive online searches and make it difficult to determine when to end their search. They also noted the extensive amount of information available about pregnancy-related problems and child diseases, which could increase their feelings of insecurity. As one participant remarked: *“Sometimes I could just keep searching … just in case there was a bad thing, I could continue to search for it sometimes. Then I felt like this: ‘What are you doing? Stop searching, so that you do not get in a bad mood’ … It was so hormonal that you kind of cried for the smallest little thing.”*

On the other hand, some participants felt that their feelings of insecurity had decreased from using digital tools to learn about other parents’ experiences with pregnancy, labor and birth, and parenthood. They described how advice from other parents was meaningful and entertaining to them, and they used this advice as guidance in their own parenting. Reading about other parents’ experiences and similar problems was reassuring, increased their sense of security, and thereby reduced their anxiety. Besides, reading about other parents’ birth experiences and watching their private birth videos helped them feel better prepared for their own birth, as one expecting mother described:

*You feel calm afterwards* [after reading about other parents’ birth experiences]. *There’s nothing you need to panic about or anything like that. Because you sort of go through the same thing and so ... Yes, that’s probably it. That you realize that other people you do not know go through the same thing ... The first year, the second year with children. It’s tough, it’s not easy.*

The participants described a lack of digital tools for which professional healthcare providers were responsible and highlighted issues such as parenting, hygiene, or illness in the child. The participants who did not speak Swedish as a first language experienced exclusion as some digital platforms were only available in Swedish and were therefore unavailable to them. As a result, they felt insecure about being unprepared for childbirth and parenthood. To access the information they needed, they had to translate the text themselves, which was frustrating and perceived as unreliable. They did not consider the information available in English as comparable as the text was significantly more limited compared with the Swedish text. One parent who was not fully fluent in Swedish described her experiences as follows: “*I thought it was a bit difficult because I had to translate everything. If it had been in English or another language it would have been very good because it is a very good website I think, a very good website… But it should be in other languages ​​as well because I actually think there are some pages, or rather videos, where others talked about their experiences with their children and they are in Swedish. So, if they could have done them in English it would have been very good. Then everyone would have felt safe no matter who they were.”* In contrast, digital tools presented in easy-to-read Swedish were supportive for participants who had newly arrived in Sweden. These participants felt that such tools were supportive in their way of identifying them as parents in a new country.

The participants described using digital tools to communicate with others, such as family, friends, or other parents whom they did not know before. By contacting other expectant and new parents, new social ties were created and friendships were established and/or maintained as well, which was considered valuable by these participants. They used digital tools, such as chat groups, to plan joint activities with their family or friends as well as to consult them in case of illness or receive parenting support. This could be understood as emotional support. One parent described this as follows: “*We write a lot of questions and we try to see each other once a week or every other week or something, to eat or go out and play on the playground or whatever as well. We send pictures when things happen, fun things or if the child is ill, or to just ask about different things. … It’s like a personal ‘home network.’”*.

The participants expressed how, when using digital tools together with their partner, their feelings insecurity decreased and their bond was strengthened. This is because they shared moments and experiences that they could talk about later. From this, the parental couple could clarify their roles during the upcoming labor and birth or how they would handle different parenting situations. The parental couples described such discussions as both entertaining and valuable. One participant described this as follows: “*What I have taken part of* [from digital tools] *has felt important to me. I have been able to put into words how I think about the birth: I think that this can be important to me. How do you think about birth, what do you think you can do? Eh… so that it has worked a bit like, like talking from the outside. … He expressed the other day that he also eh…, he said that a little jokingly, that he now feels readier than he has felt before. Because we have talked about different things in a… yes… quite practical way I think…”*.

Reading other people’s stories also stimulated feelings of insecurity and fear in relation to pregnancy, childbirth, and parenthood. The participants remarked how, by taking part in routines regarding fetal diagnostics in other countries, they were afraid and frustrated that Sweden did not offer examinations to the same extent. Furthermore, the participants expressed that access to others’ tragic pregnancy experiences increased their fear and anxiety. These experiences were detailed in texts and pictures about other parents’ experiences of miscarriage or giving birth to a sick or dead child: “*When they talked about children who were sick or ill, I used to be scared and I did not continue reading … I got worried. Even on the day I was going to give birth to my baby, I was afraid because I had read something about a child who became ill, so I was more afraid of it than the birth itself.”*

Furthermore, group discussions on digital platforms heightened feelings of insecurity among some participants about their own parenting skills. These participants doubted their ability to decisively deal with problematic situations, which in turn worsened their anxiety about being capable of managing parenthood: “*That you get impressions from others where they tell you about an issue that may be very problematic and then I become worried that my situation will also be just as problematic. … So maybe you doubt yourself, is it something I do wrong, or how should I do it differently so that it does not become like for this mother… it can cause more anxiety.”*

Some participants felt that other parents’ opinions and thoughts had great power. Sometimes, the tone of forum discussions was perceived as hostile or demeaning, which encouraged anxiety and greater insecurity about parenting. As a result, these parents distanced themselves from forum discussions.

#### Responsibility

The participants described experiences of responsibility in various ways when using digital tools related to pregnancy, childbirth, and parenthood. For example, they appreciated the digital tools (i.e., websites or applications) for which healthcare professionals (i.e., midwives, CHC nurses, or medical doctors) were responsible because they perceived the information these tools provided as more reliable. Digital tools that were recommended by healthcare professionals were considered particularly reliable by the participants. In contrast, other digital tools that were not controlled by healthcare professionals were regarded as mostly presenting personal experiences and opinions, as one participant noted: “*It gets less serious, in some way, if it is just an ordinary private person who explains. Because he or she can invent or just go by own experiences and still say that it is facts. Which can be a huge mistake if it’s not true… So of course, it feels better to me at least if it’s someone like maybe a midwife, or nurse, or someone, who tells or sort of shows something or so… eh… Yes, probably less risk that something will go wrong or be said wrong.”*

When the participants did not find accurate knowledge or reliable answers to their questions, they contacted healthcare professionals (i.e., a midwife or child healthcare nurse). In such situations, the participants experienced that digital tools were not able to replace real meetings with healthcare professionals. Real meetings facilitate non-verbal communication and feelings of safety and trustworthiness to a larger extent than digital tools, according to the participants’ narratives: *“This human contact when you meet someone, in such circumstances, when a person talks to you feel better. So, the digital tools, that is, it lacks human emotion, warmth. So that … you cannot get through the digital tools.”*

#### Knowledge

The participants mentioned using digital tools to gain knowledge about pregnancy, labor and birth, and parenthood. More specifically, they sought knowledge during early pregnancy concerning, for example, fetal development and pregnancy-related physiological changes. They also relied on digital tools to acquire knowledge about breastfeeding, physical exercise after giving birth, infant-related purchases, child rearing, ways to stimulate child development, and techniques for interacting with their child.

In their knowledge search, the participants found digital tools to be easily accessible and valuable. They appreciated information on a level considered as relevant to them since it facilitated their knowledge. From this, they were reassured to learn that their pregnancy-related physical changes, for example, were normal. This consequently diminished their feelings of insecurity and promoted calmness instead, which in turn helped them to handle their pregnancy-related issues better as well as to ensure their child’s well-being.

In addition, the knowledge gained from using digital tools helped the participants to feel more prepared for labor, both practically and mentally. Such knowledge included, for example, information about different phases of labor and associated outcomes, what to pack in their bag before admission to the labor ward, and different types of pain relief or breathing techniques to handle labor pains. They also obtained knowledge about their imminent transition to parenthood.

The participants felt it was advantageous to acquire knowledge through digital tools whenever they wanted, thereby giving them more control over their knowledge search. Just as well, they could choose among the large amount of information digitally available, which made it possible for them to tailor their knowledge search based on their individual needs:

*That I feel that I should be able to prepare myself, how it will feel to become a parent. Because it is a journey, you don’t have any idea of what it will mean to you… compared with many other things you learn. To some it might be good not to prepare, because it will be okay for them anyhow. But, for me, it has been good to read all I’ve been able to…*

For most of the participants, the credibility of the knowledge source was of crucial importance. Information provided by healthcare professionals had a “quality stamp,” whereas information from unreliable sources was reviewed with healthcare professionals. These participants noted that information derived from personal experiences lacked credibility because the source individuals did not consider the overall social picture, contextual factors, or family specifics when offering advice and support: “*So it’s a lot, it’s no… proven, or scientific, experience… it’s just experiences… yes from those private individuals… So, it is not research-related… I would rather have professional facts, from those who work… The fact that it is reliable, not only experiences, so experiences can differ very much and it is not always someone else’s experiences will be like mine.”*

## Discussion

Each pregnancy milestone can evoke anxiety concerning, for instance, the potential consequences of wrong choices or the failure to take urgent action when needed. Most of the time, expecting parents face such anxiety—and feelings of insecurity—on their own, often accompanied by the sense of lacking sufficient knowledge and/or competence. Previous literature on mothers describes parental roles and changes. Mercer’s [8] theoretical model of adaptation to the maternal role suggests that most of the mother’s role-taking activities occur after the baby’s arrival—in particular, between three and ten months after birth. This adaptation is described on three levels: biological (i.e., physical recovery for the mother and her adaptation to her baby’s growth and development), psychological (i.e., the mother’s reactions to and perceptions of motherhood), and social (i.e., changes in the mother’s own life and social surroundings). Adaptation may be hindered if the mother suffers from poor health or unresolved problems from earlier phases. Mercer stressed the importance of midwives in helping mothers adjust to the maternal role [8].

The parental adaptation to the life-changing process entering parenthood highlights the need for support and structure to experience health, exemplified when the interviewed parents described, insecurity, entering the role of becoming parents. Bearing the independent knowledge and responsibilities of the past as partners, transitioning to parenthood and shouldering the responsibilities of a family and the knowledge of what small children need. The interviews showed that digital support during this interlude of the parental transition—especially regarding insecurity, knowledge, and responsibilities—was important.

Because parents fear making mistakes due to their choices, they seek knowledge and decision support from resources that can guide their actions. The foremost guide may be the midwife or CHC nurse, but modern parents—including those interviewed in this study—also use digital tools. Previous research has described various factors that either facilitate or inhibit the parental transition [27]. Facilitating factors include having pertinent knowledge and feeling prepared for parenting, as well as access to both social and professional support. Such factors were evident in the interviews in this study, with participants describing them (i.e., social connections leading to emotional support, and digital tools recommended by professionals) as crucial to feeling that they benefited from using digital tools. Inhibiting factors were a lack of credible information or professional support, as well as anxiety and loss of control [27]. Such factors were also clear in the interviews in this study and could be understood as a source for feelings of insecurity. Thus, parents likely search for answers to their many questions as they arise, and they often conduct these searches with digital tools that provide immediate answers. Ideally, they should consult with competent and trustworthy coaches or guides, but such access is not always possible. Despite possessing a reasonable understanding of expecting parents’ psychological needs, especially during their first pregnancy, digital coaches, to the best of our knowledge, have not yet been examined in the context of pregnancy.

Therefore, our data, together with the theory of parental transition, from various theoretical paradigms, could add an existential dimension to the parental transition. Such existential dimensions of the parental transition have been described elsewhere [28, 29]. The interviewed parents’ experience of digital tools involved a digital community beyond the everyday physical world. Such communities seem to act in ways that transcend the role of facilitator or inhibitor, as in transition theory [11, 27], but are similar to communitas in a community performing the liminal phase together reintegrating into the family. Family, as a social institution, is embedded within a diversity of socially formalized and structured behaviors and ideas. The findings of the current study highlight insecurity, a concept that may describe being in-between roles, where parents search for a community with others by digital means. Therefore, the concepts of liminality and communitas could be employed in future studies on parental transitions, as discussed below.

The results from the interviews highlighted not only insecurity but also responsibility and knowledge in the search for digital guidance throughout the parental transition. As a modern social phenomenon in a digital society, searching for digital support could present a solution when everyday practice does not. As a life transition, embodying social relations, statuses as well as roles and obligations, the findings can be read as an essential need for parents, feeling safe and secure in parenthood, embodying a new social role. Digital tools may thus provide a virtual space in a reciprocal relation to the matrix of love for the newborn, built on a solid foundation of the perceived quality of a parental couple’s relationship, social support, and professional guidance.

But digital tools also presented a virtual timeline when the interviewed parents searched for knowledge any time they wanted, experiencing control over their knowledge search and the choices they made from the digital information available according to their individual needs. The search for guidance through a life change may be existential or, similar to Bell’s [21] discussion of liminality in relation to the pandemic, may concern “temporality, embodiment, intermediation, mobility, relationships, and identity” ( [21], p 81).

However, instead of reassuring parents, digital information might instead sometimes incite or increase feelings of insecurity and anxiety, and pregnancy may easily be perceived as beyond their control. This contradiction, and the possible need for support, may be challenging in everyday life and thus insecurity could be described in relation to the concept of liminality. Parental support as a contradictory, complementary search during a transitory process could thus be a search for a shared way to undergo the process of the transition, of the passage, and of the community within digital support as a communitas of social responsibility and embodied knowledge.

The interviewed parents used digital tools for social communication with family, friends, or other parents not familiar to them before. By connecting with other expectant and new parents, and appreciating these new social ties and established friendships online, the shared experiences could be interpreted as a digital communitas in the process of guidance. These feelings of belonging are in line with previous research that has found that social contextual circumstances have an impact on the parental transition [7, 9, 27, 30] and could be of importance for the professions of midwives, CHC nurses, and health workers to consider when offering support. A community undergoing such a transition together could manifest as in-person meetings or virtual meetings online. By considering the possibility of a need for a communitas between parents-to-be, further research areas may be identified.

From the context of the parental transition, the dialectic relation between family structures of past society and contemporary parenthood is in dialogue through, for example, images and norms of parenthood of families, parenthood among friends and wider society, and personal experiences of becoming a parent. Similarly, norms and ideals expressed online may contribute to how parents chose to live parenthood [31].

Easing insecurity was an aspect of the interviewed parents’ descriptions, as was the search for ways and structures rebuilt through parental transitions in need of reliable and flexible parental support. However, one part of Stephenson’s [17] descriptive critique concerns liminality as a transition and points to major life-cycle events, such as birth, focusing on the sacred. Stephensson [17] noted that theoretical comparisons between digital spheres and liminality have been made and must be treated with a great deal of skepticism. However, of interest for the current study was whether parents’ search for a digital cicerone concerning changes characteristic of the parental transition, or even life crises, was, rather than existential in a sacred sense, a dimension of liminality referring to ambivalence or chaos reminiscent of the insecurity evoked from the findings. With detachment or separation from the pre-liminal two-partner life, and a post-liminal constructive reintegration with the social structure but with new roles and responsibilities of family building and parenthood, the liminal reach for digital support may suggest an existential dimension of digital health.

Parents—likely unconsciously—seek to shift the responsibility for their concerns and choices to external resources. This shift is understandable, and it leads to another important phenomenon in this context: responsibility for one’s choices. Responsibility, whether personally managed or referred to professionals, seems to be shouldered again when parents become confident in the new parental role. Responsibility could thus be a further dimension of digital health.

By performing the transition together, by forming a family and a communitas with other parents online and in person, by shouldering responsibilities for insecurities and knowledge, those identifying the need for a digital communitas may make valuable contributions to professional practice, supporting parents in search of a reintegrated place in the social structure.

Professional digital parental support also needs to be inclusive of everyone. The interviewed parents needed digital parental support in their mother tongue, to prevent insecurity from being excluded from information, and experiences of being unprepared for childbirth and parenthood. To feel secure during the parental transition, and to enhance the possibility of communitas and feelings of belonging, it is important for digital parental support to be inclusive and open to various languages. All parents should experience being included in professional parental support for such support to be socially sustainable, which is in line with the Swedish law that mandates that patients should receive information to promote participation and consent in care [32].

Another related aspect is the possible link between liminality and the parental experience of insecurity stemming from frustration and feelings of injustice when available digital tools present the norms, ideals, and language of the majority population. Is exclusion from society a starting point for searching for a digital community, forming a communitas, to feel secure and included in the transition? This could relate to features of uncertainty and status as discussed by Underwood and Rhodes [18]. Future research could, therefore, explore the dimension of insecurity from social exclusion and how communitas, and comfort with social support, can build inclusive and sustainable relations and communities through the shared experience of parents undergoing the parental transition together. Can digital communitas be a possibility for shouldering the new responsibilities of parenthood, for building on knowledge and experiences, and for integrating them into the future life of parents?

The interviewed parents derived meaning and entertainment from advice given by other parents, which was intended to provide guidance in their own parenting. They took part in other parents’ private experiences of giving birth. Parents could experience similar problems, reassuring one another, providing safety and reducing anxiety, and preparing for birth. This may imply the need to appreciate the concept of communitas in transitory processes, and could inspire parental transition theory to include and foreground the need for social relations and community during liminality.

In a communitas-inspired framework, we assume that trustworthy pregnancy cicerones, who guide parents as partners or companions during their pregnancies and are aware of the wider community of parents, are what parents need most. This assumption leads us back to the following questions: *Do expecting parents seek information, knowledge, or reassurance in making the right decisions and/or shifting responsibility, which may diminish their insecurity? Could digital parental support tools from trustworthy resources offer the answers and security that new parents likely need?* Our study provided answers to these questions.

The interviewed parents turned to professionals when they did not find reliable answers to their questions, which has been demonstrated previously [33]. Additionally, they felt that digital tools could not replace in-person meetings with healthcare professionals, in which non-verbal communication and trustworthiness reflected secure and safe guidance. Besides, the interviewed parents valued the credibility of information sources, and healthcare professionals represented quality and reliability. This may also illustrate the dialectic relation between the structure of professional knowledgeability, the liminality of private personal experiences and stories, and feelings of insecurity.

Shifting the responsibility for expecting parents’ choices to a third party may diminish their insecurity and bring about a feeling of relief. Naturally, they expect to assess the trustworthiness of third parties so that they can rely as much as possible on such resources. Choosing a reliable resource is itself a parental choice, and the responsibility for this decision lies with parents themselves. If the quality of these resources is important to parents, understanding which parameters they consider important in their evaluation process would be useful. Given the importance of this process and its parameters, an open question remains: *How do expecting parents evaluate the quality of retrieved information and perceive the reliability of sources? Do parents and professionals have the same expectations for or perceptions of digital tools/information? When expecting parents search for information through digital channels, do they seek content, emotional support, or both?* These questions seem to be important for aligning parents’ own views of the importance of information with those of professionals in order to provide optimal guidance through a variety of informational resources.

Grounded in the above discussion, the contribution of the current study is threefold. Its contribution to the field of knowledge on expecting parents’ experiences of digital tools during parental transition is that (1) insecurity can be both eased and enhanced from using digital tools, (2) that parents could take responsibility for both their feelings of insecurity and their search for knowledge, and that (3) parents need appropriate knowledge to make the right choices and feel more secure in making these choices in the best interest of their new family.

The study’s contribution to theory is its suggestion that, in the context of becoming a parent, with its existential, life-changing dimension, transition theory may need to further study ritual theory. From the context of the current study, the concepts of liminality and communitas could inspire the development of a theory that allows for contradictions, that processes are not always linear but can be dialectic or oscillating. Even more important is the awareness that community is not only a force affecting the transition but may also be a pattern within the transition itself, one in which the transition becomes ritualized together with others.

The contribution to professions suggests that parents need professional support, for both knowledge and guidance in insecurity, both digitally and in person. However, there also seems to be a need for greater awareness that parents are involved with family, friends, and other parents, online and in person, and that these social relations with others may not only be entertaining but may also constitute a way to undergo change. Greater recognition that all kinds of parents and types of parenthood are needed to build socially sustainable parental support is also warranted.

### Strength and limitations

In the present study, the participants were limited to parents living in two regions in Sweden. However, as they were diverse in terms of age, cultural background, and parental experiences, they could be considered as representative of parents living in Sweden. In some of the interviews, a professional interpreter was used, which might have influenced the participants’ ability to feel relaxed and understood during the interviews. However, the researchers agreed that the resulting narratives were rich and that using an interpreter afforded the opportunity to include non-Swedish-speaking parents in the study. Applying two-step qualitative analysis through inductive content analysis, and thematic analysis, could have risked cementing themes in the eyes of the beholder. However, both methods are structured, and include an interpretative thread. The alternate between empirical transcripts and analyzed themes, could instead support a rigorous analysis.

## Conclusion

Parents’ experiences of using digital tools during the parental transition were that (1) insecurity can be both eased and enhanced from using digital tools, (2) they could take responsibility for both their feelings of insecurity and their search for knowledge, and (3) they needed appropriate knowledge to make the right choices and feel more secure in making these choices in the best interest of their new family. When theorizing parents undergoing a transition into parenthood, the concepts of insecurity, responsibility and knowledge may suggest a theoretical complement to transition theory. The theoretical development could thus enhance the ambiguity and adaptation to new social roles, and could be elaborated by the concept of liminality. Building ties to other parents in person and online could imply that transition theory can develop a dimension similar to communitas. To conclude, in the midst of life changes and health in a digital society, parents appear to require a digital cicerone in health care, one who can provide guidance in the parental transition.

## Data Availability

The datasets generated and analyzed during the current study are not publicly available due to limitations in the ethical permission of the study. First author should be contacted for requests.

## References

[1] Keesara S, Jonas A, Schulman K (2020). Covid-19 and Health Care’s Digital Revolution. N Engl J Med.

[2] Shin JY (2021). Designing Technologies to Support Parent-Child Relationships: A Review of Current Findings and Suggestions for Future Directions. Proc ACM Hum Comput Interact.

[3] Aksoy Derya Y (2021). Pregnancy and birth planning during COVID-19: The effects of tele-education offered to pregnant women on prenatal distress and pregnancy-related anxiety. Midwifery.

[4] Bäckström C, et al. Corrigendum: Parents' Perceptions About Future Digital Parental Support-A Phenomenographic Interview Study. Front Digit Health. 2021;3:729697. 10.3389/fdgth.2021.805357 PMC8693455 34957463

[5] Vickery M (2020). Midwives’ views towards women using mHealth and eHealth to self-monitor their pregnancy: A systematic review of the literature. Eur J Midwifery.

[6] Bäckström C (2021). Digital tools as parental support – A study protocol describing prospective development and exploration of two digital tools for parents. Front Digit Health.

[7] Schumacher K, Meleis A (1994). Transitions. A central concept in nursing. J Nurs Scholarsh.

[8] Mercer RT (1986). First-Time Motherhood: Experiences fron Teens to Forties.

[9] Linden K, Domgren N, Zaigham M, Sengpiel V, Andersson ME, Wessberg A. Being in the shadow of the unknown — Swedish women’s lived experiences of pregnancy during the COVID-19 pandemic, a phenomenological study. Women Birth. 2021;35(5):440-6. 10.1016/j.wombi.2021.09.007 PMC9364685 34602340

[10] Entsieh AA, Hallström IK (2016). First-time parents’ prenatal needs for early parenthood preparation-A systematic review and meta-synthesis of qualitative literature. Midwifery.

[11] Meleis AI (2000). Experiencing transitions: an emerging middle-range theory. Adv Nurs Sci.

[12] Lima-Pereira P, Bermúdez-Tamayo C, Jasienska G (2012). Use of the Internet as a source of health information amongst participants of antenatal classes. J Clin Nurs.

[13] Berg M (2018). Web-Based Intervention for Women With Type 1 Diabetes in Pregnancy and Early Motherhood: Critical Analysis of Adherence to Technological Elements and Study Design. J Med Internet Res.

[14] Zhao X, Basnyat I (2018). Online social support for “Danqin Mama”: A case study of parenting discussion forum for unwed single mothers in China. Comput Hum Behav.

[15] Andalibi N (2022). LGBTQ Persons’ Use of Online Spaces to Navigate Conception, Pregnancy, and Pregnancy Loss: An Intersectional Approach. ACM Trans Comput Hum Interact..

[16] Turner VW (2008). The Ritual Process: Structure and Anti-Structure.

[17] Stephenson B (2020). The Limits of Liminality: A Critique of Transformationism. Liminalities.

[18] Underwood J, Rhodes C (2018). A qualitative investigation of hospital visitor“ experiences using the analytic lens of liminality: Informing nursing practice and policy. Nurs Inquiry.

[19] Buechner B (2020). From Liminality to Communitas: The Collective Dimensions of Transformative Learning. J Transformative Educ.

[20] Laver C, et al. ‘You don’t take things too seriously or un-seriously’: Beyond recovery to liminal and liminoid possibility in a community arts and mental health project. J Commun Appl Soc Psychol. 2022;32(4):653-64.

[21] Bell G (2021). Pandemic Passages: An Anthropological Account of Life and Liminality during COVID-19. Anthropol Action.

[22] White I, McSharry M (2021). Preservice teachers’ experiences of pandemic related school closures: anti-structure, liminality and communitas. Ir Educational Stud.

[23] Gieseler C (2018). Gender-reveal parties: performing community identity in pink and blue. J Gend Stud.

[24] Bäckström C (2021). Parents’ experiences of receiving professional support through extended home visits during pregnancy and early childhood – A phenomenographic study. Front Public Health.

[25] Graneheim UH, Lundman B (2004). Qualitative content analysis in nursing research: concepts, procedures and measures to achieve trustworthiness. Nurse Educ Today..

[26] Braun V, Clarke V (2008). Using thematic analysis in psychology. Qualitative Res Psychol.

[27] Barimani M (2017). Facilitating and inhibiting factors in transition to parenthood - ways in which health professionals can support parents. Scand J Caring Sci.

[28] Gamgam Leanderz Å (2021). Parental couple separation during the transition to parenthood. Nurs Open.

[29] Prinds C, et al. Making existential meaning in transition to motherhood - A scoping review. Midwifery. 2014;30(6):733-41. 10.1016/j.midw.2013.06.021 23928210

[30] Bäckström C, Larsson T, Thorstensson S. How partners of pregnant women use their social networks when preparing for childbirth and parenthood: A qualitative study. Nordic J Nurs Res. 2021;41(1):25-33.

[31] Liechty T (2018). "It’s Just Not Very Realistic”: Perceptions of Media Among Pregnant and Postpartum Women. Health Commun.

[32] Patientlagen (2014:821). In SFS 2019:964, S. riksdag, Editor.

[33] AlJaberi H (2018). Developing Culturally Sensitive mHealth Apps for Caribbean Immigrant Women to Use During Pregnancy: Focus Group Study. JMIR Hum Factors.

[34] The World Medical Association Declaration of Helsinki, W. Ethical Principles for Medical Research Involving Human Subjects. Adopted by the 18th WMA General Assembly. 2004. Helsinki, Finland, 1964.

